# Carotid artery stenting versus no stenting assisting thrombectomy for acute ischaemic stroke: protocol for a systematic review of randomised clinical trials with meta-analyses and trial sequential analyses

**DOI:** 10.1186/s13643-016-0388-0

**Published:** 2016-12-01

**Authors:** Henrik Steglich-Arnholm, Markus Holtmannspötter, Christian Gluud, Derk Wolfgang Krieger

**Affiliations:** 1 Department of Neurology, Rigshospitalet, Copenhagen University Hospital, 2082, Rigshospitalet, Blegdamsvej 9, 2100 Copenhagen, Denmark; 2 Department of Neuroradiology, Rigshospitalet, Copenhagen University Hospital, Copenhagen, Denmark; 3 The Copenhagen Trial Unit, Centre for Clinical Intervention Research, Rigshospitalet, Copenhagen University Hospital, Copenhagen, Denmark; 4 Deptartment of Neurology Comprehensive Stroke Center, University Hospital, Zurich, Switzerland; 5 Deptartment of Neurology Mediclinic City Hospital, Stroke Unit, Dubai Health Care City, Dubai, United Arab Emirates

**Keywords:** Stroke, Thrombectomy, Acute, Carotid, Carotid occlusion, Stenting

## Abstract

**Background:**

In patients with intracranial large vessel arterial occlusion, ipsilateral extracranial carotid artery occlusions or near-occlusions pose a significant hurdle in endovascular management of acute ischaemic stroke. Stenting of the carotid lesion may be beneficial in this situation to provide a stable access for introducing catheters through the carotid lesion into the intracranial vasculature and the target occlusion. Furthermore, carotid stenting may ensure ample blood flow for wash-out of clot material and reperfusion of the ischaemic penumbral tissue. However, antiplatelet therapy administered to prevent stent thrombosis and sudden increase in blood flow after reopening of the carotid lesion may increase the risk for intracranial haemorrhagic complications. This review aims to assess the benefits and harms of carotid stenting vs. no stenting assisting thrombectomy for acute ischaemic stroke.

**Methods:**

International and regional electronic databases will be searched to identify eligible randomised clinical trials. To identify further published, unpublished, or on-going and planned trials searches of Google Scholar, Worldwide Food and Drug Administrations, Worldwide Medicines Agencies, company homepages, reference lists, conference proceedings, and the Science Citation Index cited reference search index will be conducted. Manufacturers of relevant interventional equipment, authors, colleagues, and researchers active in the field will be contacted. No language restrictions will be applied to these searches. Randomised clinical trials will be included for assessing benefits and harms and quasi-randomised studies, and observational studies will be included for assessing harms of the intervention. Meta-analyses will be performed according to the recommendations of the Cochrane Handbook for Systematic Reviews of Interventions, and Trial Sequential Analyses will be conducted to control the risk of random errors and prevent premature statements of superiority of the experimental or control intervention or premature statement of futility. The quality of the evidence will be evaluated with the Grading of Recommendations Assessment, Development, and Evaluation.

**Discussion:**

This systematic review of carotid stenting in endovascular management of acute ischaemic stroke in patients with concomitant extracranial carotid lesions and intracranial embolism will assess benefits and harms of this intervention and assesses whether carotid stenting should be encouraged or avoided in acute ischaemic stroke and identify targets for further research.

**Systematic review registration:**

PROSPERO CRD42016033346

**Electronic supplementary material:**

The online version of this article (doi:10.1186/s13643-016-0388-0) contains supplementary material, which is available to authorized users.

## Background

### Description of the condition

Acute ischaemic stroke is the leading cause of acquired long-term disability and the fourth most common cause of death [1]. The severity of acute ischaemic stroke varies from minor focal neurological deficits over life-threatening hemispheric syndromes to death. Due to the high oxygen requirement of the brain tissue, expeditious management is crucial for reversal of ischaemia and successful salvage of the tissue at risk [2]. Large intracranial emboli cause severe ischaemic stroke with poor outcome and poor response to medical therapy alone due to the large clot burden [3, 4]. A particular harmful configuration of large vessel occlusions is the carotid artery occlusion or near-occlusion in combination with intracranial embolism. This configuration is suggested to be the cause of acute ischaemic stroke in up to 20 to 30% of patients with large vessel occlusions [5–8]. The carotid occlusion or near-occlusion is caused by an arterial dissection or atherosclerotic plaque. It releases an often large clot into the intracranial vasculature causing severe stroke symptoms. Usually, carotid occlusions or near-occlusions can be compensated haemodynamically via the circle of Willis [9], but not in the case of an embolus lodged in the middle cerebral artery [10].

Administration of intravenous recombinant tissue plasminogen activator (iv-rtPA) is currently the recommended first-line treatment for acute ischaemic stroke if it can be administered within 4.5 h of symptom onset [11, 12]. However, in patients suffering moderate to severe stroke from acute large vessel occlusions, iv-rtPA is often ineffective [4, 13]. Cohort studies suggest that iv-rtPA administration alone only leads to clinical improvement in 20 to 30% of patients with concomitant extracranial carotid and intracranial occlusions [13–16]. Carotid endarterectomy is not the preferred option, since surgery would only address the extracranial carotid lesion without access to the intracranial occlusion. Furthermore, open surgery is relatively contraindicated with recent iv-rtPA administration, as open surgery has a high complication rate in the very urgent phase of acute stroke [17], and is not advocated to repair carotid dissections [18].

Endovascular therapy with mechanical thrombectomy or intra-arterial thrombolysis of large intracranial occlusions have long been considered a possible adjuvant to medical therapy although initial randomised controlled trials failed to reveal clear benefits [19–21]. However, in the past year, five randomised controlled trials have shown superior outcomes of endovascular therapy compared with medical therapy alone [7, 8, 22–25]. This leads to thrombectomy of large intracranial occlusions being recommended in the American Heart Association guidelines for acute ischaemic stroke therapy with the highest evidence (class I, level of evidence A) [26]. The primary target of endovascular therapy in patients with carotid lesions and concomitant intracranial embolism is removal of the intracranial clot material. Endovascular therapy has the advantage of being able to access the intracranial thrombus either directly through the ipsilateral carotid lesion or indirectly via collateral vessels [27]. The indirect access via contralateral vessels is technically challenging and depends on favourable anatomy of the circle of Willis. The direct access is more straightforward but bares the risks with penetrating the wire through a carotid occlusion or near-occlusion unable to predict passage within the true lumen resulting in dissection of the vessel wall as well as dislodgement of thrombotic material distal to the carotid lesion.

### Description of the intervention

The concerns of the direct access approach in endovascular management of acute ischaemic stroke can to some extent be compensated by carotid stent-assisted angioplasty [28]. A carotid stent can easily be provided through the catheters used for mechanical thrombectomy [10, 29–36]. However, introduction into the carotid lesion may pose an obstacle if the carotid artery is occluded or severely stenotic. In this case, the carotid lesion may need to be balloon pre-dilated with a balloon catheter to ensure adequate lumen for traversal of the stent through the carotid lesion [10, 29–36]. Carotid stents are mostly self-expanding which means they expand to a pre-specified diameter when subjected to the heat inside a vessel. However, some carotid lesions are so dense that the carotid stent needs balloon post-dilatation to ensure adequate lumen inside and flow through the stent.

To prevent in-stent thrombosis, antithrombotic therapy is needed. To our knowledge, no guideline exists on the optimal antithrombotic regimen for carotid stenting in endovascular management of acute ischaemic stroke, and patients are treated on a patient-by-patient basis [37]. Most centres seem to favour administration of mono- or dual antiplatelet therapy immediately after stent placement and continue with dual antiplatelet therapy after intracerebral haemorrhage has been excluded after the procedure [10, 29, 33, 35, 36].

### How the intervention might work

Carotid stenting may be beneficial in acute ischaemic stroke treatment because deployment of a stent with or without angioplasty establishes immediate patency of the carotid lesion preventing vessel recoil and secures continuous catheter access to the intracranial vessels. It stabilises and protects the endothelium preventing iatrogenic dissection of the vessel wall. Furthermore, acute stenting ensures ample blood flow to the intracranial vasculature, especially in case of contralateral carotid lesions or unfavourable anatomy of the circle of Willis, and may assist intracranial recanalisation [37]. Other advantages of acute carotid stenting include acute prevention of recurrent thrombus formation and embolism from the carotid lesion, which is suggested to occur in up to 16% of patients within 24 h [38], and avoidance of a subacute procedure to prevent recurrent ischaemic events [17, 39].

Carotid stenting in the acute ischaemic stroke setting is, however, not without concerns. Immediate dual antiplatelet therapy already administered during the procedure is required to prevent acute stent thrombosis [40]. Potent aggressive antiplatelet therapy may increase the risk of haemorrhagic stroke as well as procedural bleeding complications [41, 42]—especially following recent iv-rtPA administration [43]. Furthermore, increased cerebral blood flow, seen in patients with recanalisation of chronic carotid occlusions or near-occlusions, may induce the cerebral hyper-perfusion syndrome and risk intracerebral haemorrhage [44]. Finally, if met with difficulties, preparatory carotid stenting may delay intracranial revascularisation [32, 33].

### Why it is important to do this review?

To our knowledge, only observational studies have assessed this important topic [37]. All of these patient series seem to report reasonable benefits and safety [10, 29–36]. A systematic review will provide a thorough assessment of the evidence for this intervention. Because of the before mentioned risks of carotid stenting in acute ischaemic stroke, this review is important to assess whether carotid stenting in endovascular therapy is beneficial and safe or whether carotid stenting should be avoided in the acute ischaemic stroke setting whenever possible.

### Objectives

The aims of this study are to assess the benefits and harms of acute extracranial carotid artery stenting versus no stenting in patients with acute ischaemic stroke caused by an extracranial carotid occlusion or near-occlusion in association with thrombectomy for concomitant intracranial embolism.

## Methods

### Study registration

This protocol follows the Preferred Reporting Items for Systematic Reviews and Meta-Analyses Protocol 2015 (PRISMA-P, Additional file 1) and is registered on PROSPERO with CRD42016033346.

### Criteria for considering studies for this review

#### Types of studies

This review will include randomised clinical trials for assessments of benefits and harms and quasi-randomised studies and observational studies for assessments of harms of the intervention.

#### Types of participants

Participants will be adults (≥18 years) with acute ischaemic stroke caused by a carotid artery occlusion or near-occlusion with concomitant ipsilateral embolism to a major intracranial vessel. Intracranial and extracranial occlusions or near-occlusions need to be identified on CT angiography, MR angiography, or duplex sonography and confirmed on digital subtraction angiography. Participants need to be treated within 6 h of symptom onset.

#### Types of interventions

The experimental group will be patients who were randomised to undergo extracranial carotid stenting within the same procedure as the intracranial thrombectomy. Carotid stenting may be performed before or after intracranial thrombectomy using any endovascular stent device.

The comparison group will be patients who were randomised to avoid carotid stent deployment. The comparison group may undergo carotid angioplasty without stenting, patent artery occlusion of the carotid artery after successful thrombectomy, or no carotid intervention within the same procedure as intracranial thrombectomy.

Co-interventions will be allowed if they are used equally in both the intervention and comparison groups. However, co-interventions (such as pre- or post-dilatation of the carotid artery to facilitate stent deployment) that are generally regarded as a pre-requisite for the intervention are accepted as an integrated part of the experimental intervention. Antiplatelet therapy is administered within the endovascular procedure following stent deployment in most patients of the intervention group while this is not the case for the comparison group and patients with successful carotid stenting have tightly controlled and treated mean arterial blood pressure typically not exceeding 100 mmHg after the procedure. These co-interventions will be allowed as an integrated part of the experimental intervention although they are not used equal in both groups of a trial.

#### Types of outcome measures

Outcomes will be assessed after 3 months (primary outcome time point) and at maximal follow-up.

### Primary outcomes

- All-cause mortality
- Dependent clinical appearance measured as a score on the modified Rankin Scale of 3 or more
- Serious adverse events defined as any untoward medical occurrence that is life threatening, results in death or persistent or significant disability, or any other event that may have jeopardised the participant or require intervention to prevent it [45]

### Secondary outcomes

- Quality of life
- Non-serious adverse events

### Exploratory outcomes

- Haemorrhagic complications (symptomatic/asymptomatic)
- Periprocedural embolic events into new territory
- Recurrent ipsilateral ischaemic stroke during follow-up

### Search methods for identification of studies

#### Electronic searches

The searches will be performed in cooperation with a Trials Search Coordinator from the Copenhagen Trial Unit and will include the following electronic databases:

- Cochrane Central Register of Controlled Trials (http://www.thecochranelibrary.com/view/0/index.html)
- PubMed (http://www.ncbi.nlm.nih.gov/pubmed)
- Embase (http://www.embase.com)
- Stroke Trials Directory (www.strokecenter.org/trials)
- Clinicaltrials.gov (www.clinicaltrials.gov)
- Current controlled trials (www.controlled-trials.com)
- World Health Organisation’s International Clinical Trials Registry Platform (http://apps.who.int/trialsearch/Default.aspx)

#### Regional databases

- African Index Medicus (http://indexmedicus.afro.who.int/)
- Australasian Medical Index (http://www.nla.gov.au/australasian-medical-index, http://www.informit.com.au/health.html)
- Chinese Biomedical Literature Database (CBM) (in Chinese) (http://www.imicams.ac.cn/)
- Index Medicus for the Eastern Mediterranean Region (http://www.emro.who.int/his/vhsl/)
- PASCAL (https://www.ebscohost.com/academic/pascal)
- IndMED (http://indmed.nic.in/)
- KoreaMed (http://www.koreamed.org/SearchBasic.php)
- LILACS (http://lilacs.bvsalud.org/en/)
- Index Medicus for the South-East Asia Region (http://imsear.hellis.org/)
- Panteleimon (http://www.panteleimon.org/maine.php3)
- Western Pacific Region Index Medicus (http://www.wprim.org/)

The initial draft for the electronic database search presented in MEDLINE (Ovid SP) format:

1. exp Stents/
2. ((carotid and stent*) or CAS).mp.
3. exp Thrombectomy/
4. (thrombectom* or thrombolys*).mp.
5. 1 or 2
6. 3 or 4
7. 5 and 6
8. exp Brain Ischemia/
9. exp Carotid Stenosis/
10. (stroke or isch*emi* or (carotid and (occlusion or near-occlusion or stenos* or obstruct*)) or apople*).mp.
11. 8 or 9 or 10
12. 7 and 11

### Searching other resources

To identify further published, unpublished, or on-going and planned trials, the following measures will be taken:

- Search Google Scholar
- American Food and Drug Administration (FDA)
- European Medicines Agency (EMA)
- Health Canada (http://www.hc-sc.gc.ca/index-eng.php)
- Australian Therapeutic Goods Administration (TGA https://www.tga.gov.au/)
- China Food and Drug Administration (CFDA http://eng.sfda.gov.cn/)
- Brazilian Health Surveillance Agency (ANVISA http://portal.anvisa.gov.br/)
- Mexican Federal Commission for the Protection against Sanitary Risk (COFEPRIS http://www.cofepris.gob.mx/Paginas/Idiomas/Ingles.aspx)
- Argentinian National Administration of Drugs, Foodstuffs and Medical Technology (ANMAT http://www.anmat.gov.ar/principal_en.asp)
- Columbian National Food and Drug Surveillance Institue (INVIMA https://www.invima.gov.co/)
- Thailand Food and Drug Administration (TFDA http://www.fda.moph.go.th/eng/index.stm)
- Taiwan Food and Drug Administration (TFDA http://www.fda.gov.tw/EN/)
- Singapore Health Sciences Authority (HAS http://www.hsa.gov.sg/content/hsa/en.html)
- Japanese Pharmaceuticals and Medical Devices Agency (PMDA http://www.pmda.go.jp/english/index.html)
- South Korea Ministry of Food and Drug Safety (MFDS http://www.mfds.go.kr/eng/index.do)
- Indian Central Drugs Standard Control Organisation (http://cdsco.nic.in/forms/contentpage1.aspx?lid=1423)
- Home pages of companies producing devices for the interventions
- Screening reference lists of relevant trials
- Contact manufacturers of relevant interventional equipment
- Contact authors, colleagues, and researchers active in the field
- Identify and hand-search the proceedings of relevant conferences
- Use the Science Citation Index Cited Reference search for forward tracking of relevant references

No language restrictions will be applied to the searches, and translations of potentially relevant non-English language papers will be obtained.

### Data collection and analysis

#### Selection of studies

Two review authors (Henrik Steglich-Arnholm and Derk W. Krieger) will independently screen titles and abstracts identified by the searches. Henrik Steglich-Arnholm will be responsible for obtaining full paper copies of eligible trials and translation into English if necessary. Henrik Steglich-Arnholm, Derk W. Krieger, and Marcus Holtmannspötter will assess the full paper copies for inclusion into the review. In case of disagreements regarding which papers to obtain full paper copies of and which trials to include a solution will be found by discussion between the review authors with Christian Gluud arbitrating.

### Data extraction and management

Two review authors (Henrik Steglich-Arnholm and Markus Holtmannspötter) will independently extract data from each eligible trial onto a standard designed data extraction form. Review authors will not be blinded to journal or institution. Disagreements will be resolved by discussion among all authors.

The following data will be extracted from included studies:

- First author
- Country of origin
- Trial design
- Inclusion and exclusion criteria
- Number of participants
- Patient characteristics
- Details of endovascular interventions used (including methods for intraarterial clot removal/lysis, antegrade or retrograde stenting approach, angioplasty before/after stent placement, stents used, etc.)
- Details of pharmacological therapy used (e.g., antiplatelet agent, dose, route of administration, intravenous or intraarterial thrombolysis, etc.)
- Follow-up period
- Primary and secondary outcomes
- Adverse events
- Diagnostic criteria used for acute ischaemic stroke (including whether MRI diffusion and perfusion mismatch, CT-angiography or CT perfusion were used to identify eligible patients)
- Co-interventions used (including type of anaesthesia)
- Anatomy of arterial occlusions
- Time interval from stroke onset to procedure start
- Time interval from stroke onset to intracranial recanalisation
- Time interval from procedure start to recanalisation
- Numbers of patients in each group with outcomes
- Patients lost to follow-up

### Assessment of risk of bias in included studies

For randomised clinical trials the following domains will be assessed for risks of bias: allocation sequence generation; allocation concealment; blinding of patients and personnel; blinding of assessors; incomplete outcome reporting; selective outcome reporting; industry bias; and other apparent biases [46]. Domains will be assessed according to these definitions:

Allocation sequence generation

- Low risk of bias: sequence generation is achieved using computer generated random numbers or a random number table, or similar.
- Uncertain risk of bias: the trial is described as randomised, but the method of sequence generation is not specified.
- High risk of bias: the sequence generation method is not, or may not be, random. Quasi-randomised studies, those using dates, names, or admittance numbers in order to allocate patients, are inadequate and are excluded for the assessment of benefits but not for assessing harms.

Allocation concealment

- Low risk of bias: allocation is controlled by a central and independent randomisation unit, sequentially numbered, opaque and sealed envelopes, or similar, so that intervention allocations cannot be foreseen in advance of or during enrolment.
- Uncertain risk of bias: the trial is described as randomised but the method used to conceal the allocation is not described, so that intervention allocations may be foreseen in advance of or during enrolment.
- High risk of bias: if the allocation sequence is known to the investigators who assigned participants or if the study is quasi-randomised. Quasi-randomised studies are excluded for the assessment of benefits but not for assessing harms.

Blinding of participants and personnel

- Low risk of bias: it is mentioned that both the participants and personnel providing the interventions were blinded, and the method of blinding is described, so that knowledge of allocation is prevented during the trial.
- Uncertain risk of bias: it is not mentioned if the trial was blinded, or the trial is described as blinded, but the method or extent of blinding is not described, so that knowledge of allocation was possible during the trial.
- High risk of bias: the trial is not blinded, so that the allocation was known during the trial.

Blinded outcome assessment

- Low risk of bias: outcome assessment was carried out blinded for all relevant outcomes, and the method of blinding is described, so that knowledge of allocation was prevented.
- Uncertain risk of bias: blinding of outcome assessment is not described, or the outcome assessment is described as blinded, but the method of blinding is not described, so that knowledge of allocation was possible.
- High risk of bias: outcome assessment is not blinded, so that the allocation was known to outcome assessors.

Incomplete outcome data

- Low risk of bias: the numbers and reasons for dropouts and withdrawals in all intervention groups are described, or if it is specified that there are no dropouts or withdrawals.
- Uncertain risk of bias: the report gives the impression that there have been no dropouts or withdrawals but this is not specifically stated.
- High risk of bias: the number of or reasons for dropouts and withdrawals are not described.

Selective outcome reporting

- Low risk of bias: pre-defined or clinically relevant and reasonably expected outcomes are reported on.
- Uncertain risk of bias: not all pre-defined or clinically relevant and reasonably expected outcomes are reported on, or are not reported on fully, or it is unclear whether data on these outcomes are recorded or not.
- High risk of bias: one or more clinically relevant and reasonably expected outcomes are not reported on; data on these outcomes are likely to have been recorded.

Industry bias

- Low risk of bias: the trial appears to be free of industry influence that could put it at risk of bias. The trial did not receive direct financial support, products for use in the trial, or funding for conduct of trial tasks (e.g., analysis of data) from any parties that might had conflicting interest (e.g., a drug or a device manufacturer).
- Uncertain risk of bias: the trial may or may not be free of industry bias that could put it at risk of bias or did not provide financial disclosure statements.
- High risk of bias: the trial is not considered free of industry involvement.

Other bias

- Low risk of bias: the trial appears to be free of other components that could put it at risk of bias.
- Uncertain risk of bias: the trial may or may not be free of other components that could put it at risk of bias.
- High risk of bias: there are other factors in the trial that could put it at risk of bias, e.g., authors have conducted other trials on the same topic, etc.

Trials with low risk of bias assessments in all of the above mentioned domains are considered as having low risk of bias. Trials with one or more domains that are unclear or high risk of bias will be considered trials with high risk of bias [47].

Observational studies will be evaluated according to the ACROBAT-NRSI tool for both cohort-type studies and case-control-type studies [48].

### Measures of treatment effect

Odds ratio with 95% confidence interval will be reported for dichotomous outcomes. Mean differences or standardised mean differences with 95% confidence interval will be reported for continuous outcomes.

For each trial, a table describing the types of serious adverse events reported will be presented.

### Unit of analysis issues

#### Dealing with missing data

In the case of missing data, the authors of the original trials and studies will be contacted in attempt to obtain further details.

#### Intention-to-treat analyses

Incomplete or missing outcome data will be imputed according to extreme case scenarios [49]:

- ‘Worst-best’ case scenario—extreme case analysis favouring the experimental intervention. All the missing event-data from the experimental group, but none of the missing event-data from the control group will be imputed as a bad outcome.
- ‘Best-worst’ case scenario—extreme case analysis favouring the control. None missing event-data from the experimental group, but all of the missing event-data from the control group will be imputed as a bad outcome.

#### Assessment of heterogeneity

Heterogeneity will be assessed quantitatively with Cochran Q and *I* ^2^ statistics as well as qualitatively. Furthermore, fixed- and random-effects models will be compared (see below).

#### Assessment of reporting biases

Funnel plots will be used for assessing reporting and other types of bias. These plots will be assessed for asymmetry qualitatively. If more than 10 studies are included in the meta-analyses, funnel plots will also be assessed for asymmetry quantitatively as follows.

#### Continuous outcomes with intervention effects measured as mean differences

Funnel plot asymmetry will be assessed with a linear regression approach suggested by Egger et al. [50]. In this graphical approach, the mean difference of each study is divided by its standard error and plotted against the inverse of its standard error. Smaller studies should have larger standard errors and intercept trough (0,0). If this line does not intercept in zero, then there is a risk of a small-study effect and possible bias. Furthermore, the slope of this line will indicate the size and direction of the intervention effect.

#### Dichotomous outcomes with intervention effects measured as odds ratios

For dichotomous outcome measures, the above mentioned method is not feasible for estimating a small-study effect because the estimates of the effect size and of the standard error are correlated.

Therefore, if there is a low between-study heterogeneity (*τ* ^2^ < 0.1), a modified version of the linear regression method mentioned above will be used. In this version, an efficient score is plotted against a score variance which is not correlated. These estimates are calculated from number of events and participants in the control and intervention group and the total number of participants [51].

On the other hand, if there is not low between-study heterogeneity (*τ* ^2^ ≥ 0.1), an arcsine transformation of event-fractions can stabilise variances (study precision) and a heterogeneity parameter is introduced in the regression model. This allows testing for small-study effects with sufficient power even when substantial heterogeneity is present [52].

### Data synthesis

Meta-analyses will be performed according to the recommendations of the Cochrane Handbook for Systematic Reviews of Interventions [46]. For the statistical analyses, Review Manager 5.3 (Copenhagen: The Nordic Cochrane Centre, The Cochrane Collaboration, 2014), SAS software version 9.4 (SAS Institute Inc., Cary, NC, USA), and Trial Sequential Analysis version 0.9 (Copenhagen Trial Unit, Centre for Clinical Intervention Research, Rigshospitalet, Copenhagen, Denmark; www.ctu.dk/tsa) will be used.

#### Trial sequential analyses

Conventional meta-analysis methods usually only assess statistical significance of the intervention effect from the accrued evidence in a review and lack assessment of the amount of evidence behind these effect estimates. Such assessment is available through Trial Sequential Analysis methods. Trial Sequential Analyses will control the risk of random errors due to sparse data and potential repetitive analyses of data and prevent premature statements of superiority of the experimental or control intervention or premature statement of futility [53, 54]. Trials will be included in the sequential analysis one by one and ultimately checked if predefined two-sided significance testing- or futility boundaries are crossed and if the diversity-adjusted required information size is reached [55]. The diversity-adjusted required information size for dichotomous outcomes will be calculated for the primary and secondary outcomes on the basis of the proportion of outcomes in the control group, a relative risk reduction of 20% as well as the relative risk in randomised clinical trials with low risks of bias (if such trials can be identified), a type I error of 2.5%, a type II error of 20% (80% power), and the diversity of the meta-analysis [56]. The type I error risk will be based on Jakobsen and colleagues’ correction for multiplicity [57]. Finally, Trial Sequential Analyses will establish if sufficient studies are identified in order to make firm conclusions on the safety and efficacy of carotid artery stenting in acute ischaemic stroke.

#### Assessments

Intervention effects will be assessed with both random-effects model meta-analyses [58] and fixed-effect model meta-analyses [59]. The more conservative point estimate of the two (the estimate closest to no effect or the estimate with the widest confidence interval) will be used [57]. Furthermore, each of the eight steps proposed by Jakobsen and colleagues [57] will be addressed to validate the results from the meta-analyses.

The conclusion will primarily be based on results from trials with low risk of bias in all domains and on the primary outcomes after 3 months.

### Subgroup analysis

The following subgroup analyses are planned:

- Trials with low risk of bias compared to trials with high risk of bias
- Trials assessing preparatory carotid stenting compared to trials assessing carotid stenting after thrombectomy
- Trials compared according to type and dosage of antiplatelet regimen used
- Trials assessing the combination of intravenous thrombolysis with single or dual antiplatelet therapy administered during the intervention compared to trials using no concomitant thrombolysis and antiplatelet therapy
- Trials assessing pre- and/or post-stenting angioplasty in patients with carotid stenting
- Trials assessing level of anaesthesia (general anaesthesia or conscious sedation) for patients with carotid stenting as well as patients without carotid stenting

### ‘Summary of findings’ table

To minimise incorrect interpretations of findings and recommendations, a ‘summary of findings’ table [46] will be constructed for all outcomes using GRADEprofiler (https://gradepro.org/). The Grading of Recommendations Assessment, Development, and Evaluation (GRADE) approach appraises the quality of a body of evidence based on the extent to which one can be confident that an estimate of effect or association reflects the item being assessed [60]. The quality of a body of evidence considers within-study risk of bias, the indirectness of the evidence, heterogeneity of the data, imprecision of effect estimates, and risk of publication bias.

## Discussion

This review will assess the benefits and harms of extracranial carotid stenting versus no stenting in acute ischaemic stroke patients treated with thrombectomy for ipsilateral intracranial occlusions. Endovascular therapy was recently implemented in the American Heart Association guidelines for treating acute ischaemic stroke caused by an intracranial large vessel occlusion [26]. However, our preliminary searches have suggested only limited evidence for acute carotid stenting during endovascular management of a large intracranial occlusion in observational studies. A thorough assessment of the evidence for carotid stenting in this setting is needed to guide clinicians in this therapeutic conundrum.

## Additional file

Additional file 1: Preferred Reporting Items for Systematic review and Meta-Analysis Protocols 2015 (PRISMA-P). Recommended items to address in a protocol for a systematic review. (DOC 36.9 kb)

## Abbreviations

iv-rtPA: Intravenous recombinant tissue plasminogen activator
CT: Computer tomography
MR: Magnetic resonance
